# Global Estimates of COVID‐19 Morbidity and Mortality: A Cohort Study and Mathematical Model Analysis

**DOI:** 10.1111/irv.70154

**Published:** 2025-12-01

**Authors:** Houssein H. Ayoub, Hiam Chemaitelly, Laith J. Abu‐Raddad

**Affiliations:** ^1^ Mathematics Program, Department of Mathematics and Statistics, College of Arts and Sciences Qatar University Doha Qatar; ^2^ Infectious Disease Epidemiology Group, Weill Cornell Medicine‐Qatar Cornell University Doha Qatar; ^3^ Department of Population Health Sciences, Weill Cornell Medicine Cornell University New York New York USA; ^4^ Department of Public Health, College of Health Sciences, QU Health Qatar University Doha Qatar; ^5^ College of Health and Life Sciences Hamad bin Khalifa University Doha Qatar

**Keywords:** cohort study, epidemiology, fatality, mathematical model, SARS‐CoV‐2, severity

## Abstract

**Background:**

The true extent of the severity and fatality caused by the COVID‐19 pandemic remains uncertain. This study provides an approximate estimate of the global disease burden from the pandemic.

**Methods:**

A cohort study followed the Qatari population from the onset of the pandemic to March 18, 2024, to estimate the age‐specific incidence rates of severe, critical, and fatal COVID‐19, classified according to World Health Organization criteria. A mathematical model then utilized these rates to generate regional and global estimates of COVID‐19 severity and fatality.

**Results:**

The incidence rate of any severe, critical, or fatal COVID‐19 throughout the pandemic was 1.13 (95% CI: 1.07–1.19) per 1000 person‐years, while that of fatal COVID‐19 alone was 0.11 (95% CI: 0.09–0.13) per 1000 person‐years. Globally, the number of severe, critical, or fatal COVID‐19 cases was estimated at 61.9 million (95% UI: 55.0–69.9 million), while the number of fatal COVID‐19 cases alone was estimated at 11.3 million (95% UI: 7.8–17.4 million). Both estimates showed large regional variations. Most severe, critical, and fatal COVID‐19 cases occurred during the pre‐Omicron phase of the pandemic.

**Conclusions:**

The COVID‐19 pandemic had a profound global impact on morbidity and mortality, emphasizing the critical need for preparedness for future pandemics.

## 1 Introduction

The coronavirus disease 2019 (COVID‐19) pandemic, caused by the severe acute respiratory syndrome coronavirus 2 (SARS‐CoV‐2), has inflicted widespread morbidity and mortality [1, 2, 3]. The pandemic has also caused economic losses and societal disruptions due to mandated social and physical distancing measures aimed at curtailing virus transmission [4]. However, the true morbidity and mortality impact of this pandemic remains uncertain due to variations in reporting practices and testing availability across different countries [2, 3, 5]. Overwhelmed healthcare systems during peak surges and deaths at home may have also contributed to inaccuracies in death certification, with some COVID‐19‐related deaths being attributed to other causes [1, 2]. The lack of application of standardized criteria for defining and reporting COVID‐19 severity and mortality has further exacerbated discrepancies in reported data [6].

The World Health Organization (WHO) established a classification system for COVID‐19 case severity [7], criticality [7], and fatality [8]. Qatar appears to be the only country to have consistently implemented this standardized WHO classification at a national level to assess the severity of COVID‐19 cases from the onset of the pandemic to recent time [6, 9]. Trained medical personnel evaluate the severity of COVID‐19 cases using a protocol that is applied to every hospitalized COVID‐19 patient [6, 9, 10, 11]. Importantly, COVID‐19‐associated hospitalizations, some of which may reflect hospitalization with COVID‐19 rather than because of COVID‐19, are not used as a proxy for COVID‐19 severity, as these have limitations in accurately capturing the true severity of COVID‐19 [9, 12, 13].

This situation contrasts with that in other countries. Definitions of severe and fatal COVID‐19 varied widely across settings and shifted over time [6, 9, 13, 14, 15]. Early in the pandemic, many health systems adopted criteria derived from WHO guidance, typically defining severe disease by oxygen impairment, respiratory distress, or need for intensive care [7, 8, 9, 14]. In practice, however, thresholds differed by local capacity, and hospitalization often served as a proxy for severity [9]. During surges, resource constraints may have further distorted classifications, as not all clinically severe cases were consistently identified [16].

Fatal COVID‐19 was likewise inconsistently defined [14]. Some systems counted any death occurring within a fixed period after a positive test, while others required COVID‐19 to be formally certified as the underlying cause of death [9, 14]. Although WHO promoted standardized attribution, implementation varied considerably across countries [14]. Differences in certification practices, diagnostic capacity, and health information systems further complicated cross‐country comparisons of severe and fatal COVID‐19 [6, 9, 12, 13, 14].

Against this background, Qatar's consistent application of rigorous, standardized WHO criteria throughout the pandemic offers a unique opportunity to estimate global COVID‐19 morbidity and mortality. In this study, we first estimated the incidence rates of severe [7], critical [7], and fatal [8] COVID‐19 by age group in the Qatari population, as per WHO classification, across the three pandemic phases: the pre‐Omicron phase, the first Omicron wave, and the post‐first Omicron wave. We then extrapolated and applied these rates to the population of each country by age group and summed them to arrive at global estimates for COVID‐19 morbidity and mortality, after factoring variations in access to and quality of healthcare across countries. While acknowledging limitations due to variations in circulating SARS‐CoV‐2 variants, comorbidity rates, and vaccination and other intervention rollouts across countries, this method offers a reasonable and feasible approach to estimate, albeit crudely, COVID‐19 morbidity and mortality in the absence of more precise methods.

## 2 Methods

This article integrates results from two studies to provide global estimates of COVID‐19 severity and mortality. The first, a retrospective cohort study, follows the methodology of a previous work [6] and updates it to estimate age‐specific incidence rates of severe [7], critical [7], and fatal [8] COVID‐19 cases within the Qatari population. Notably, only Qatari citizens were included, while Qatar has a large expatriate population [17]. This is because the expatriate population, composed primarily of young male craft and manual laborers [18, 19], might not reflect a typical national population's health profile [20, 21]. The Qatari population, on the other hand, offers a representative mix of health statuses.

The second study utilizes a mathematical modeling approach. It applies the incidence rates obtained from the first study to the age‐structured populations of countries with over one million inhabitants. By summing these results, the study generates regional and global estimates of COVID‐19 severity and mortality. The methodologies of both studies are described in detail below.

### 2.1 Cohort Study Analysis

#### 2.1.1 Data Sources

The study analyzed data across three distinct pandemic phases defined by the broad type of circulating SARS‐CoV‐2 variants. The pre‐Omicron phase spanned from the first documented infection on February 28, 2020, to December 18, 2021 [22], just before the onset of the first Omicron wave [23]. The first‐Omicron phase encompassed the first (very large) Omicron wave, lasting from December 19, 2021, to February 28, 2022 [23, 24]. Finally, the post‐first Omicron phase covered the period from March 1, 2022, to the study's conclusion on March 18, 2024, during which various Omicron subvariants dominated incidence [24, 25, 26, 27, 28]. Although individual SARS‐CoV‐2 variants and subvariants differed in severity throughout the pandemic [5, 6, 29, 30, 31, 32, 33], the objective of this study was to estimate the average severity within each defined period, thereby capturing the collective impact of the variants circulating during that phase.

COVID‐19 data were retrieved from Qatar's integrated, nationwide digital health information platform (Supporting Information: Section S1). This platform includes all SARS‐CoV‐2‐related records, encompassing COVID‐19 hospitalizations, deaths, and both polymerase chain reaction (PCR) and medically supervised rapid antigen testing, irrespective of location or facility (Supporting Information: Section S2).

SARS‐CoV‐2 testing was extensive in Qatar until October 31, 2022, with nearly 5% of the population being tested every week, primarily for routine purposes such as screening or meeting travel‐related requirements [11, 34]. Subsequently, testing rates decreased, with less than 1% of the population being tested per week [27]. Most SARS‐CoV‐2 infections were diagnosed through routine testing rather than symptomatic presentation (Supporting Information: Section S1) [11, 34].

COVID‐19 vaccination was almost exclusively carried out using mRNA vaccines [27, 35, 36] and was administered throughout the pandemic according to United States Food and Drug Administration‐approved protocols. Further details on Qatar's population and COVID‐19 databases have been previously published [11, 17, 21, 34, 37, 38, 39, 40].

#### 2.1.2 Severe, Critical, and Fatal COVID‐19

As part of the national standardized protocol for COVID‐19 case assessment, all hospitalized patients with confirmed SARS‐CoV‐2 infection (whether in acute care or intensive care unit (ICU) beds) underwent an infection severity assessment every 3 days following WHO guidelines. This assessment continued until the patient was discharged or died. Trained medical personnel, independent of the study investigators, reviewed individual patient charts to classify cases as severe [7], critical [7], or fatal [8] (Supplementary Appendix, Section S3). Patients with severe illness were typically admitted to acute care units, with occasional placement in ICU beds as a precautionary measure. Conversely, critically ill patients were always admitted to ICU beds.

The incidence of severe, critical, and fatal COVID‐19 cases was defined as the first assessment during hospitalization indicating the respective severity level. For newly admitted patients, a severe or critical assessment occurring at least 30 days after their previous hospital discharge was considered a new and independent diagnosis.

This study retrieved COVID‐19 severity (severe, critical, and fatal) records for every documented SARS‐CoV‐2 infection or reinfection since the pandemic's onset. Reinfection was defined as a documented infection occurring ≥ 90 days after a previous one to avoid misclassifying prolonged positivity as reinfection [23, 41, 42]. Patients who developed severe, critical, or fatal COVID‐19 after infection (or reinfection) were classified based on the worst outcome—death [8] followed by critical disease [7] and then severe disease [7] (Supporting Information: Section S3). The date of outcome incidence was set as the day of the positive SARS‐CoV‐2 test that documented the infection leading to severe, critical, or fatal COVID‐19.

#### 2.1.3 Cohort Follow‐up

This cohort study followed 312,876 Qatari individuals born on or before February 28, 2020, the start of the pandemic in Qatar. The national SARS‐CoV‐2 testing database, which should encompass the entire Qatari population due to widespread testing mandates and routine testing practices (Supporting Information: Section S1) [6, 11, 21, 34], was used to identify this population. Within the study period ending March 18, 2024, this cohort underwent a total of 3,000,390 tests, averaging 9.6 tests per person.

The study followed this cohort from the study start date (February 28, 2020) until one of the following events occurred: documented SARS‐CoV‐2 infection/reinfection resulting in COVID‐19 death, death from causes unrelated to COVID‐19, or the administrative end of follow‐up (March 18, 2024).

#### 2.1.4 Statistical Analysis

The study characterized the national cohort by calculating frequency distributions and measures of central tendency. The incidence rate of any severe, critical, or fatal COVID‐19 outcome, both overall and by age group, was determined by dividing the number of episodes by the total person‐years contributed by the participants in the cohort. A Poisson log‐likelihood regression model with the Stata 18.0 *stptime* command was employed to estimate the incidence rate and its corresponding 95% confidence interval (CI). This approach was also used to estimate the incidence rate of specifically fatal COVID‐19 outcomes. The analysis assessed these incidence rates across the entire follow‐up period as well as for the three distinct pandemic phases defined above.

Standardized incidence rates, adjusted to the global population age structure, were also calculated and reported. The global age structure was based on the WHO World Standard Population [43, 44].

All statistical analyses were conducted using Stata/SE version 18.0 (StataCorp LLC, College Station, TX, USA).

### 2.2 Mathematical Modelling Analysis

#### 2.2.1 Global and Regional Estimation of Severe, Critical, or Fatal COVID‐19

The number of severe, critical, and fatal COVID‐19 cases, along with specifically the number of fatal COVID‐19 cases, was estimated for each country and territory with a population exceeding 1 million using the following expressions applied for each pandemic phase and throughout the pandemic:

$$\begin{array}{l} \text{Number of severe},\text{critical},\text{or fatal COVID}‐19\;\text{cases}=\sum_{a}\left(\lambda_{a}^{\text{Severe},\text{critical},\text{or fatal}}\times N_{a}\times T\right)\\ \text{Number of fatal COVID}‐19\;\text{cases}=\sum_{a}\left(\lambda_{a}^{\text{Fatal}}\times N_{a}\times T\right) \end{array}$$

Here, $\lambda_{a}^{\text{Severe},\text{critical},\text{or fatal}}$ and $\lambda_{a}^{\text{Fatal}}$ represent the incidence rate of severe, critical, or fatal COVID‐19 and the incidence rate of fatal COVID‐19, respectively, among each age group a as estimated in the cohort analysis above for each pandemic phase and throughout the pandemic. $N_{a}$ denotes the population size in each age group and in each country, extracted from the United Nations World Population Prospects database [45]. T denotes the follow‐up duration: 4.2 years overall, divided into 2.0 years in the pre‐Omicron phase, 0.2 years in the first‐Omicron phase, and 2.0 years in the post‐first Omicron phase.

COVID‐19 mortality can be affected by access to and quality of healthcare in a given country. Countries with lower healthcare access and quality may experience higher COVID‐19 mortality rates. For instance, a critical COVID‐19 case can result in a COVID‐19 death if an ICU is not available. The Global Burden of Disease study's age‐specific Healthcare Access and Quality (HAQ) Index [46] was used for each country to adjust for these variations across countries. The above expression for the number of fatal COVID‐19 cases for each age group in each country was adjusted by multiplying it by the following ratio:

$$\mathrm{HAQ}\;\text{ratio for each}\;\mathrm{age}\;\text{group}=\frac{\text{Highest}\;\mathrm{HAQ}\;\text{Index globally for each}\;\mathrm{age}\;\text{group}}{\text{Country specific}\;\mathrm{HAQ}\;\text{Index for each}\;\mathrm{age}\;\text{group}\;}$$

HAQ Index provides a scoring system ranging (theoretically) from 0 to 100 for healthcare access and quality for three age brackets in each country: 0–14, 15–64, and 65–74 years [46]. Figure S1 shows the age‐stratified HAQ ratio for each country across the WHO regions.

The HAQ adjustment used the highest global HAQ Index as a reference instead of Qatar's HAQ Index. This decision was based on the fact that Qatar's HAQ Index was estimated using data from before 2019, which may not accurately reflect the current situation. Qatar has experienced rapid healthcare development in recent years, including a major expansion of healthcare resources and access before the World Cup 2022 [17, 18, 21, 47, 48], and its Human Development Index has been rapidly growing [49]. Qatar's response to the COVID‐19 pandemic was also notably strong, featuring a well‐resourced healthcare system and the rapid deployment of healthcare workers from other countries [17, 18, 21, 47, 48].

However, a sensitivity analysis was conducted to assess the impact of using Qatar's HAQ Index as the reference. In addition, a second sensitivity analysis tested two alternative methods for adjusting for healthcare access and quality in each country: (1) applying the square root of the age‐stratified HAQ ratio instead of the ratio itself and (2) applying the age‐stratified HAQ ratio inflated by a logarithmic addition of its value. These approaches bracket two extremes—one that underestimates and one that inflates the effect of healthcare access and quality.

The study generated estimates for COVID‐19 severity and mortality for 159 countries and territories, encompassing virtually the entire global population [45]. Results are reported both globally and regionally, categorized by the six World Health Organization (WHO) regions: African (AFRO), Region of the Americas (AMRO), Eastern Mediterranean (EMRO), European (EURO), South‐East Asia (SEARO), and Western Pacific (WPRO).

#### 2.2.2 Uncertainty Analysis

Since the model is expressed as an algebraic function of the included variables—written directly as mathematical equations combining the input variables in a fixed, explicit way, each with its own 95% CI—the 95% uncertainty interval (UI) for each estimated outcome was derived by propagating the 95% CIs of the input variables, including the age‐specific incidence rates. For only estimates of COVID‐19 deaths, the 95% CIs of the HAQ Index for each country were also propagated.

This method for the uncertainty analysis integrates the model input uncertainties to produce the widest possible UI for each estimated outcome in the study. The 95% UI for the proportion of cases by pandemic phase was calculated by applying the high and low UI bounds for both the numerator and denominator of the ratios.

All mathematical modeling analyses were conducted in MATLAB R2020b (MathWorks, Natick, MA, USA).

## 3 Oversight

Weill Cornell Medicine‐Qatar and Hamad Medical Corporation Institutional Review Boards approved this retrospective study with a waiver of informed consent. All methods were performed in accordance with the relevant guidelines and regulations. The study was reported following the Strengthening the Reporting of Observational Studies in Epidemiology (STROBE) guidelines (Table S1).

## 4 Results

### 4.1 Cohort Analysis

**FIGURE 1:**
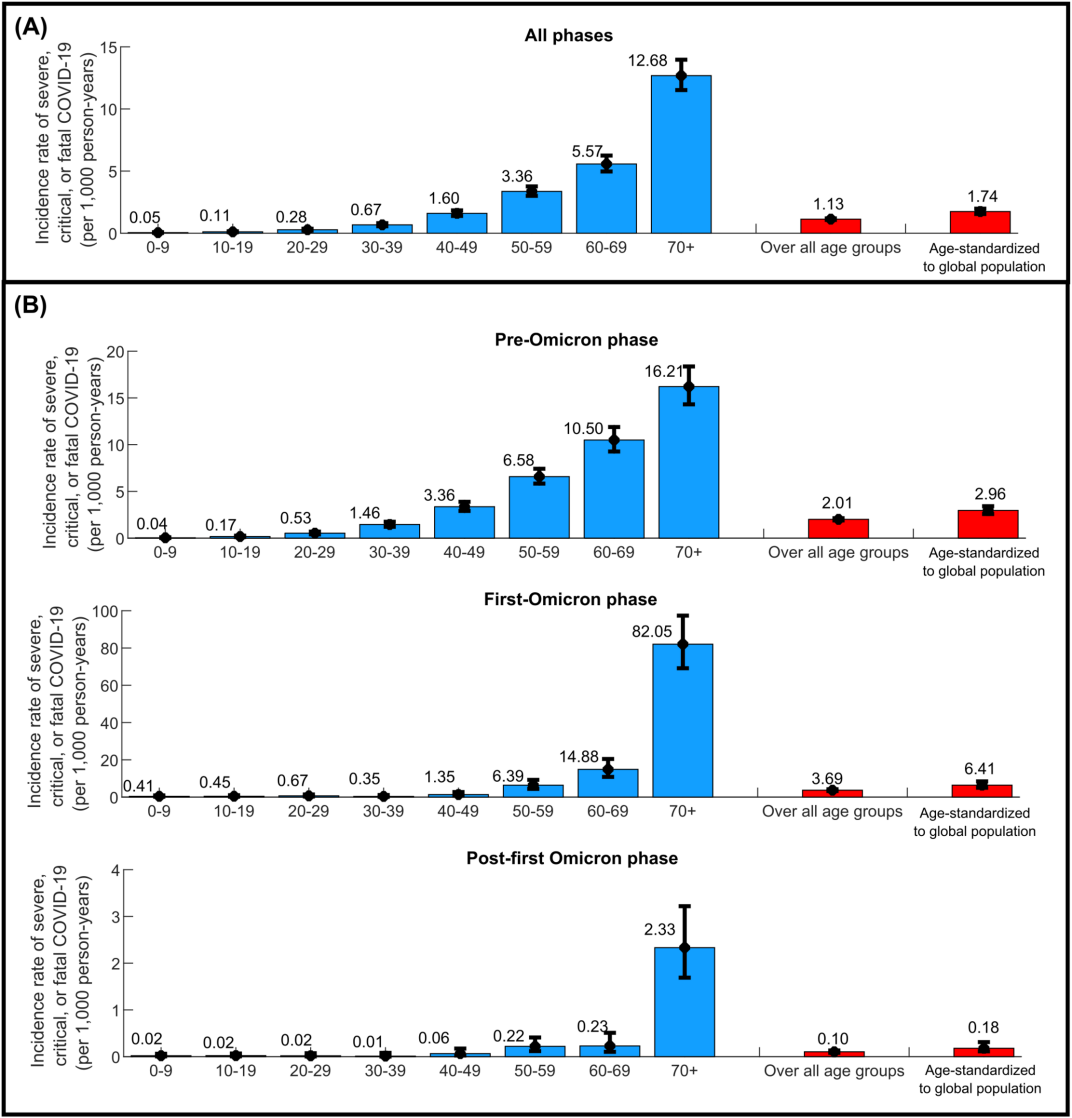
Incidence rate of any severe, critical, or fatal COVID‐19 per 1000 person‐years: (A) overall throughout the pandemic and (B) stratified by the pre‐Omicron, first‐Omicron, and post‐first Omicron phases. Age‐standardized rates to the global population were calculated using the World Health Organization World Standard Population (2000–2025) [43, 44].

Table S2 provides the baseline characteristics of the cohort study population. Figure 1 shows both the age‐stratified and overall incidence rates of severe, critical, or fatal COVID‐19 among the study cohort. The incidence rate of severe, critical, or fatal COVID‐19 showed a very strong age dependence. The overall incidence rate throughout the pandemic was estimated at 1.13 (95% CI: 1.07–1.19) per 1000 person‐years. During the pre‐Omicron phase, the first‐Omicron phase, and the post‐first Omicron phase, the overall incidence rates were 2.01 (95% CI: 1.90–2.13), 3.69 (95% CI: 3.24–4.21), and 0.10 (95% CI: 0.08–0.13) per 1000 person‐years, respectively. When standardized to the global population age structure, the overall incidence rate across the pandemic was estimated at 1.74 (95% CI: 1.54–1.97) per 1000 person‐years.

**FIGURE 2:**
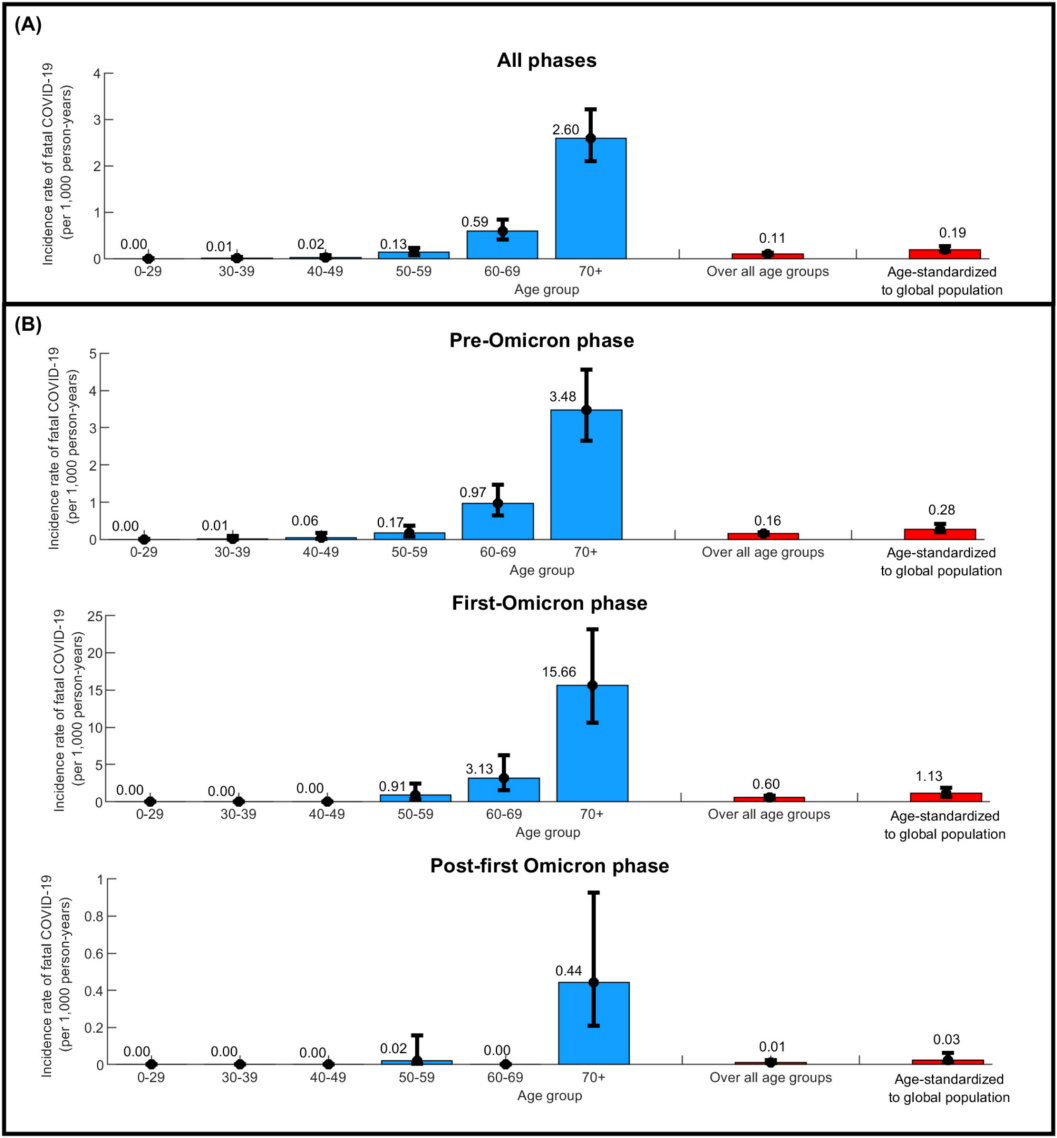
Incidence rate of fatal COVID‐19 per 1000 person‐years: (A) overall throughout the pandemic and (B) stratified by the pre‐Omicron, first‐Omicron, and post‐first Omicron phases. Age‐standardized rates to the global population were calculated using the World Health Organization World Standard Population (2000–2025) [43, 44].

Figure 2 shows both the age‐stratified and overall incidence rates of fatal COVID‐19 among the study cohort. Incidence rate of fatal COVID‐19 showed a very strong age dependence. The overall incidence rate throughout the pandemic was estimated at 0.11 (95% CI: 0.09–0.13) per 1000 person‐years. During the pre‐Omicron phase, the first‐Omicron phase, and the post‐first Omicron phase, the overall incidence rates were 0.16 (95% CI: 0.13–0.19), 0.60 (95% CI: 0.44–0.83), and 0.01 (95% CI: 0.01–0.03) per 1000 person‐years, respectively. When standardized to the global population age structure, the overall incidence rate across the pandemic was estimated at 0.19 (95% CI: 0.15–0.27) per 1000 person‐years.

### 4.2 Estimates for Severe, Critical, or Fatal COVID‐19 Cases and COVID‐19 Deaths

**FIGURE 3:**
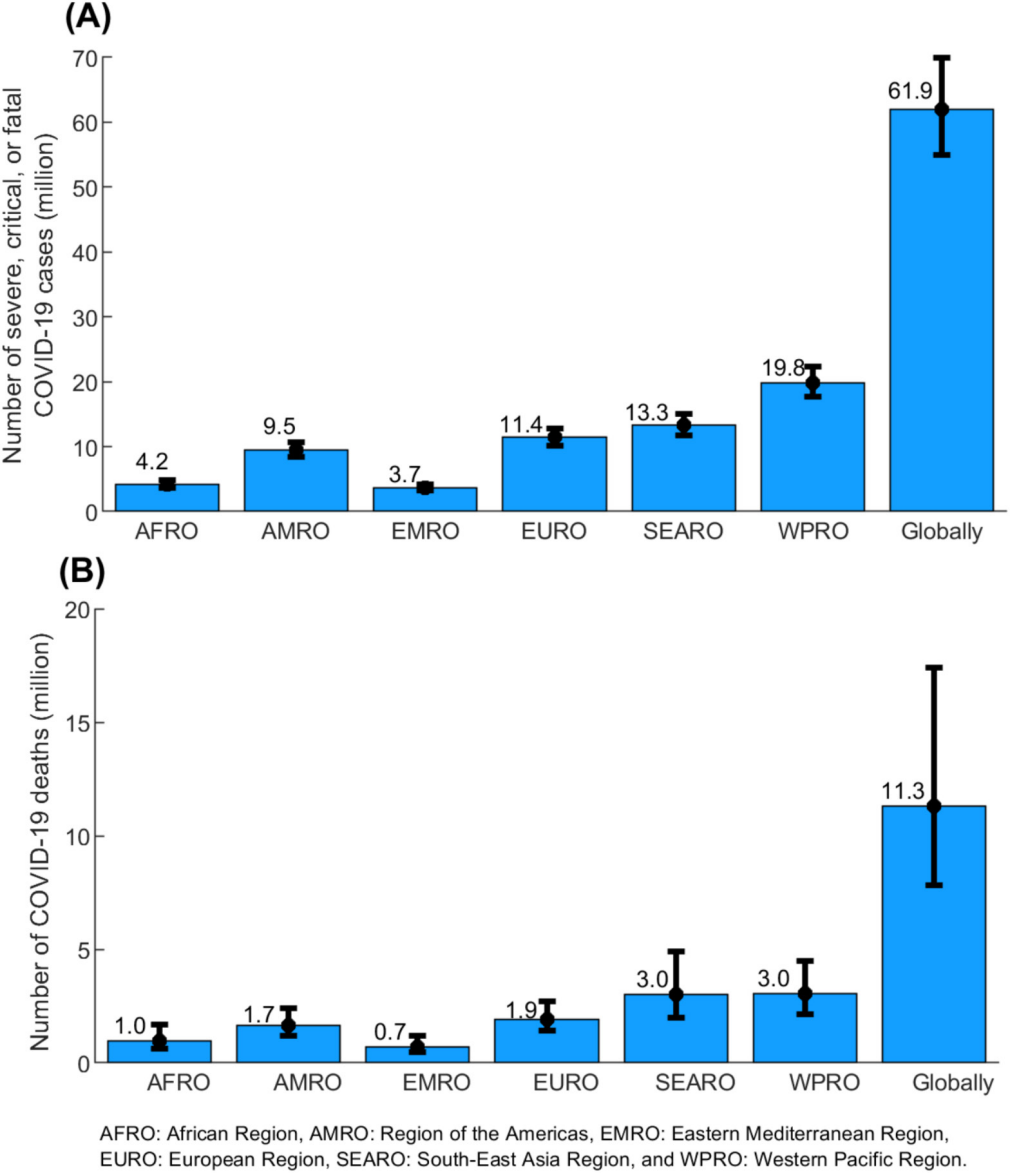
Model‐estimated (A) number of severe, critical, or fatal COVID‐19 cases, and (B) number of COVID‐19 deaths, globally and across WHO regions throughout the pandemic.

Figure 3A shows the estimated number of severe, critical, or fatal COVID‐19 cases globally and by WHO region throughout the pandemic. Globally, the number of severe, critical, or fatal COVID‐19 cases was estimated at 61.9 million (95% UI: 55.0–69.9 million). There was large variation by region. The number of severe, critical, or fatal COVID‐19 cases was lowest in EMRO at 3.7 million (95% UI: 3.2–4.2 million), followed by AFRO at 4.2 million (95% UI: 3.6–4.8 million), AMRO at 9.5 million (95% UI: 8.5–10.7 million), EURO at 11.4 million (95% UI: 10.2–12.8 million), SEARO at 13.3 million (95% UI: 11.8–15.1 million), and highest in WPRO at 19.8 million (95% UI: 17.7–22.3 million).

Figure 3B shows the estimated number of COVID‐19 deaths globally and by WHO region throughout the pandemic. Globally, the number of COVID‐19 deaths was estimated at 11.3 million (95% UI: 7.8–17.4 million). There was large variation by region. The number of COVID‐19 deaths was lowest in EMRO at 0.7 million (95% UI: 0.5–1.2 million), followed by AFRO at 1.0 million (95% UI: 0.6–1.7 million), AMRO at 1.7 million (95% UI: 1.2–2.4 million), EURO at 1.9 million (95% UI: 1.4–2.7 million), SEARO at 3.0 million (95% UI: 2.0–4.9 million), and highest in WPRO at 3.0 million (95% UI: 2.2–4.5 million).

### 4.3 Number and Proportion of Cases by Pandemic Phase

**FIGURE 4:**
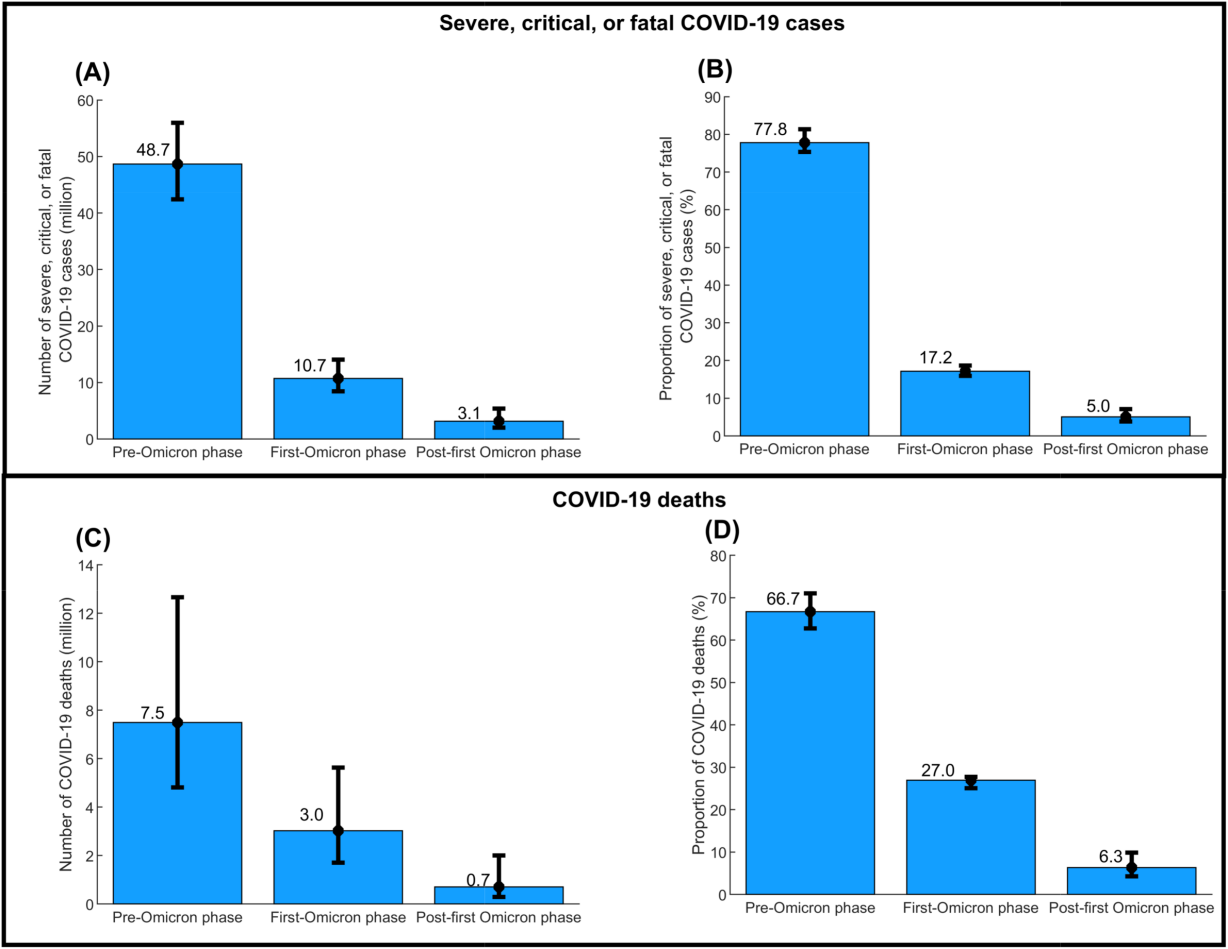
Model‐estimated global (A) number and (B) proportion of severe, critical, or fatal COVID‐19 cases, and global (C) number and (D) proportion of COVID‐19 deaths, during the pre‐Omicron phase, first‐Omicron phase, and post‐first Omicron phase.

Figure 4A shows the estimated number of severe, critical, or fatal COVID‐19 cases globally by pandemic phase. The number dropped drastically from 48.7 million (95% UI: 42.4–56.0 million) during the pre‐Omicron phase to 10.7 million (95% UI: 8.4–14.1 million) during the first‐Omicron phase and 3.1 million (95% UI: 2.0–5.4 million) during the post‐first Omicron phase. The proportion of all cases that occurred during the pre‐Omicron phase, first‐Omicron phase, and post‐first Omicron phase was 77.8% (95% UI: 74.2%–80.3%), 17.2% (95% UI: 15.9%–18.7%), and 5.0% (95% UI: 3.8%–7.1%), respectively (Figure 4B).

Figure 4C shows the estimated number of COVID‐19 deaths globally by pandemic phase. The number dropped drastically from 7.5 million (95% UI: 4.8–12.7 million) during the pre‐Omicron phase to 3.0 million (95% UI: 1.7–5.6 million) during the first‐Omicron phase and 0.7 million (95% UI: 0.3–2.0 million) during the post‐first Omicron phase. The proportion of all COVID‐19 deaths that occurred during the pre‐Omicron phase, first‐Omicron phase, and post‐first Omicron phase was 66.7% (95% UI: 62.4%–70.7%), 27.0% (95% UI: 25.1%–27.8%), and 6.3% (95% UI: 4.3%–9.9%), respectively (Figure 4D).

### 4.4 Comparison Between Model‐Estimated and Reported COVID‐19 Deaths

**FIGURE 5:**
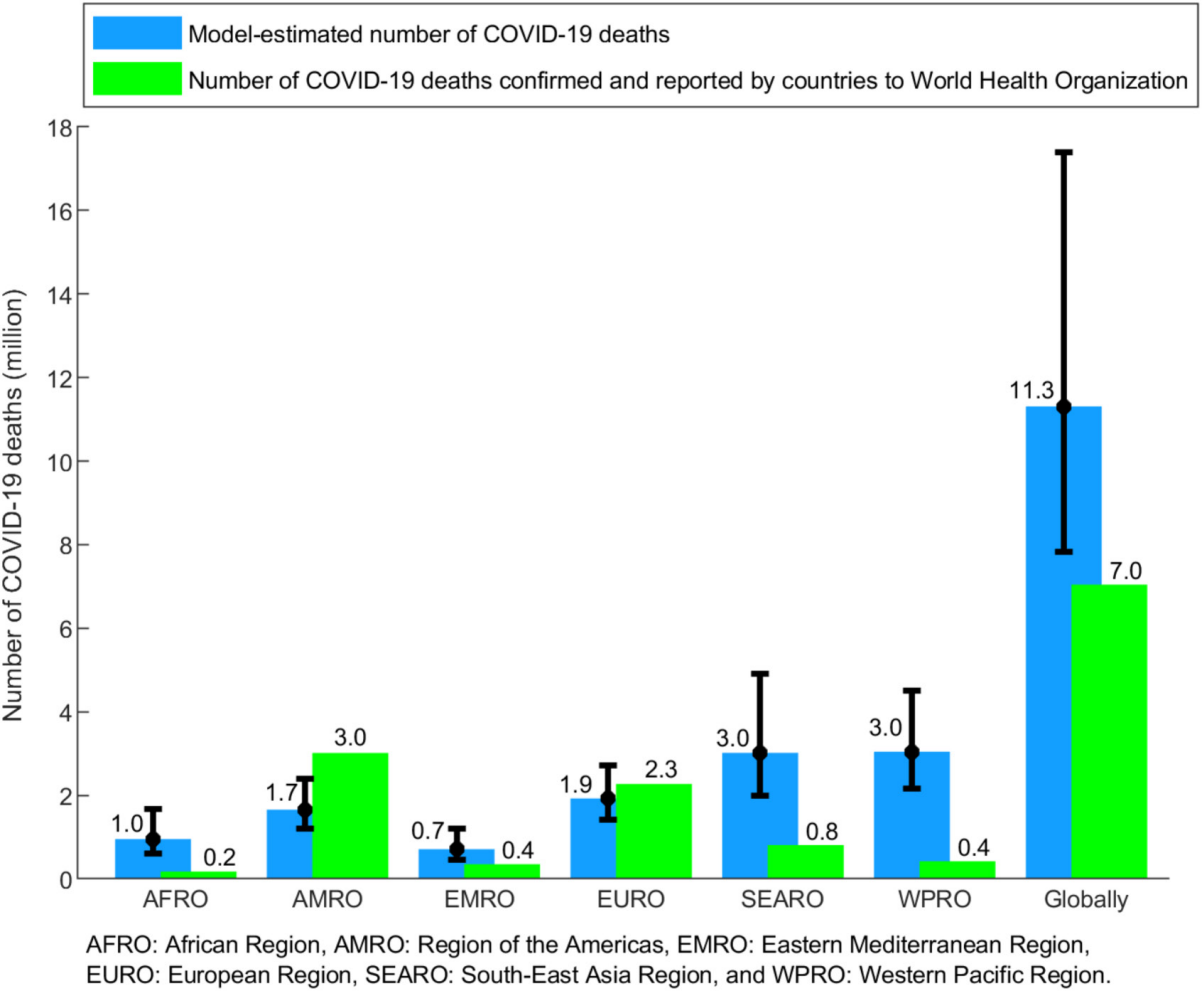
Comparison between the model‐estimated number of COVID‐19 deaths throughout the pandemic and the number of COVID‐19 deaths reported by countries to the World Health Organization, globally and by World Health Organization region.

Figure 5 compares the model‐estimated and reported global death tolls to WHO from COVID‐19, along with a regional breakdown. Globally, the model estimated 11.3 million (95% CI: 7.8–17.4 million), whereas countries reported nearly 7.0 million deaths to the WHO.

The regional breakdown reveals variations. The EURO region aligns closely with the model's estimates. However, reported deaths in the AFRO, EMRO, SEARO, and WPRO regions are considerably lower than the model's estimates. Conversely, the AMRO region is the only one where reported deaths are considerably higher than the model's estimate.

### 4.5 Sensitivity Analyses for COVID‐19 Deaths

Figure S2 illustrates the comparison between COVID‐19 death estimates from the main analysis and those from the sensitivity analysis, where Qatar's HAQ Index is used as a reference instead of the highest global HAQ Index. The sensitivity analysis showed similar results but with overall lower estimates for COVID‐19 deaths, though both estimates had overlapping uncertainty intervals. In the main analysis, the estimated number of global COVID‐19 deaths was 11.3 million (95% UI: 7.8–17.4 million), whereas in the sensitivity analysis, it was 9.0 million (95% UI: 6.2–13.9 million).

Similarly, the second sensitivity analysis, which tested two alternative approaches to adjusting for healthcare access and quality—one underestimating and one inflating the effect—yielded results consistent with the main analysis, again with overlapping uncertainty intervals (Figure S3).

## 5 Discussion

The results suggest that the COVID‐19 pandemic caused approximately 62 million severe or critical cases and around 11 million deaths. These estimates underscore the profound global impact of the pandemic, with substantial morbidity and mortality concentrated within a short period of about two years. More than two‐thirds of severe, critical, and fatal cases occurred during the first two years of the pandemic, while only a minority were observed after the pre‐Omicron phase.

While the COVID‐19 pandemic has demonstrably caused substantial morbidity and mortality, the estimates presented here are lower than projections made during the early stages of the pandemic [50], but broadly consistent with our own early projections [3] and within the range of other assessments, including WHO's estimate of excess mortality associated with the pandemic [2], noting the differences in what is being measured—COVID‐19–specific deaths in our analysis versus excess mortality in the WHO estimates.

Public health interventions such as vaccination campaigns, social and physical distancing measures, and improved case management likely contributed to this reduced disease burden [51, 52, 53, 54]. The emergence of the Omicron variant, associated with lower severity [29, 30], may have further mitigated the overall impact. However, these results suggest that the intrinsic severity of the virus itself might have been less than initially feared.

Indeed, assuming the observed incidence rates for fatal COVID‐19 and that 50% of the Qatari population was infected with SARS‐CoV‐2 before vaccination (natural infection)—a reasonable assumption based on local epidemiology [17, 18, 19, 54, 55]—the infection fatality rate (the proportion of all infections, including undiagnosed, asymptomatic, and mild cases, that result in death) is estimated roughly at 0.09%. When standardized to the global population age structure, the infection fatality rate is estimated at 0.16%. These estimates are substantially lower than those reported early in the pandemic but are largely consistent with our early pandemic estimate [3] and with late‐pandemic figures [56, 57, 58].

The estimated number of global COVID‐19 deaths was about 60% higher than that of the reported deaths to the WHO. This discrepancy reflects that the model‐derived figures for COVID‐19 deaths in several regions exceeded the WHO‐reported deaths, particularly in regions consisting primarily of lower and middle‐income countries. In these regions, resource limitations, such as testing, may have curtailed the ability to confirm COVID‐19 as the cause of death [2, 59]. Reporting of COVID‐19 deaths may also have been hindered by factors such as the quality of filling and coding of death certificates and the location of death (hospital, nursing home, or at home) [60]. In the resource‐rich EURO region, the model estimates were consistent with WHO‐reported deaths, suggesting that the difference found in lower‐income countries is due to underreporting.

While underreporting may explain discrepancies in some regions, the WPRO region presents a different scenario. Here, the model estimates for COVID‐19 deaths are substantially larger than the WHO‐reported deaths. This discrepancy might largely be due to China's zero‐COVID policy, which delayed exposure to large waves until after widespread vaccine rollout [61, 62].

In the AMRO region, the model appeared to underestimate the number of COVID‐19 deaths, but the reported number of deaths may have exceeded the true number of deaths due to classifying deaths with COVID‐19 as COVID‐19 deaths, even when COVID‐19 was not the actual cause of death per strictly the WHO criteria [8]. For example, in the United States, COVID‐19 deaths were reported if COVID‐19 was listed on the death certificate as either an underlying or contributing cause of death [60, 63]. This differs significantly from the WHO definition [8], leading to an overestimation of COVID‐19 deaths.

The study findings should be interpreted in the context of the inherent difficulty of attributing deaths directly to COVID‐19. Mortality frequently results from the interplay of COVID‐19 with pre‐existing conditions, making it challenging to establish whether COVID‐19 was the underlying cause or a contributing factor [64]. Comorbidities strongly shape COVID‐19 outcomes by amplifying viral, inflammatory, and post‐acute pathological processes, while simultaneously diminishing physiological reserve [64]. This interplay complicates efforts to disentangle the direct effects of COVID‐19 from those of coexisting illnesses [64]. Such challenges are a key reason why several global assessments, including those by WHO [2], have relied on excess all‐cause mortality rather than COVID‐19–specific mortality as a benchmark of pandemic impact [1, 2, 65, 66]. A distinctive contribution of this study is the estimation of deaths directly attributable to COVID‐19, derived using rigorous WHO‐defined criteria applied consistently across the pandemic.

Whereas our analysis focused on COVID‐19–specific deaths, excess mortality captures also unrecognized COVID‐19 deaths as well as indirect deaths caused by healthcare disruptions and broader societal effects [2]. Our estimates therefore represent a conservative baseline, while excess mortality estimates provide an upper bound of the pandemic's true mortality burden. Indeed, existing global excess mortality estimates [1, 2, 65, 66] exceed those presented here. Considered together, these complementary approaches delineate the plausible range of pandemic impacts. Future work could build on our estimates by integrating observed excess mortality from countries with robust vital registration systems, thereby accounting individually for each of the direct and indirect consequences of the pandemic.

This study has limitations. The analysis focused on severe, critical, and fatal COVID‐19 cases as defined by WHO criteria [7, 8]. However, this classification might underestimate the true disease burden. Some hospitalizations, while necessary, may not have met the WHO criteria for severity [9]. The spectrum of COVID‐19 severity may have also changed over time [67, 68, 69, 70, 71], potentially encompassing milder forms that were not captured by the WHO criteria but still required hospitalization [6, 9, 71]. In addition, the study did not examine post‐acute sequelae or Long COVID, which represent an important component of the overall burden of disease.

The study applied a specific method to adjust for the effect of healthcare access and quality on COVID‐19 mortality, but this approach has not been formally validated for this purpose. Two sensitivity analyses tested alternative versions of the adjustment—one underestimating and the other inflating the effect of healthcare access and quality—and produced results with overlapping uncertainty intervals compared with the main analysis. This suggests that different adjustment methods may not materially alter the findings, largely because the adjustment has the greatest impact in countries with substantially lower healthcare access and quality than in high‐income settings. These countries also tend to have younger populations, which are less affected by fatal COVID‐19 and therefore contribute less to the overall estimates.

Given the small size of the Qatari population aged over 70 years, individuals in this age group were combined into a single category, and statistically precise further‐stratified incidence rate estimates for severity and fatality could not be generated. However, severity and fatality incidence rates vary substantially within those older than 70 [56, 72, 73, 74]. This broad categorization may have limited the representativeness of our estimates for the elderly and their applicability to populations with a larger share of older individuals, such as many high‐income countries.

Vaccination coverage varied globally in terms of vaccine type, timing, uptake of the primary series, and booster rollouts, and thus, fatality and severity rates observed in Qatar may not be generalizable to other countries. Nonetheless, COVID‐19 severity and fatality were most concentrated among those aged over 50 years, as shown here and in studies from other settings [1, 2, 56, 65, 66, 72, 73, 74]. Countries with large elderly populations are predominantly high‐income, where vaccine access and coverage were broadly comparable to those in Qatar. By contrast, countries with delayed vaccine access generally have younger populations and were therefore less affected, in relative terms, by severe and fatal outcomes. While differences in vaccine access and coverage represent an important limitation, they may not substantially alter the estimates presented in this study to change the conclusions drawn.

In general, extrapolating even rigorously derived age‐specific COVID‐19 severity and mortality rates from one country to others introduces inherent limitations. Outcomes are shaped by differences in healthcare resources, standards of care, case management practices, comorbidity profiles, socioeconomic conditions, genetic factors, circulating variants, and the nature, scale, and timing of interventions. These factors constrain the extent to which infection metrics, even when carefully estimated in one setting, can be generalized elsewhere.

Despite these limitations, several factors may mitigate their impact. Exposure to the virus has been extensive in most countries, often with similar circulating variants. The inherent biology of the virus likely played a primary role in its severity and mortality, with age being a decisive factor, as demonstrated in our analyses (Figures 1 and 2). All analyses presented in this study were adjusted for age and accounted for variations in healthcare access and quality.

It is also important to emphasize that the aim of this study is not to provide definitive figures, but rather approximate estimates that improve understanding of the global disease burden and epidemiology of COVID‐19 through a distinct approach. By leveraging unique datasets, we utilized consistent, rigorous, and standardized WHO criteria for severity and fatality across the entire pandemic. Qatar appears to be the only country to have implemented this classification nationally throughout the pandemic [6, 9], offering a rare opportunity to estimate global morbidity and mortality impact. Estimating the global disease burden of COVID‐19 is inherently challenging and subject to unavoidable limitations in all methods, and thus, a full picture of the pandemic's impact remains incomplete.

Nevertheless, the findings presented here add to a more comprehensive understanding of the pandemic's toll on global health and provide data to inform preparedness for future pandemics. Importantly, when considered alongside other studies conducted at different stages of the pandemic, a converging picture emerges that brackets the likely impact. Our estimates fall within this range but toward the lower end, suggesting that the pandemic's toll and the intrinsic severity of the virus were less than initially feared. Even so, SARS‐CoV‐2 caused extensive severity and fatality as it entered and circulated in human populations.

In conclusion, the study highlights the profound global impact of the COVID‐19 pandemic, with an estimate of 62 million severe or critical cases and 11 million deaths. However, these figures fall below initial projections, in part due to the public health interventions and potentially a lower intrinsic severity of the virus itself, particularly following the emergence of the Omicron variant. The findings underscore the critical need to prepare for future pandemics, considering their toll on health as well as their broader impacts on societies and economies.

## Author Contributions

**Houssein H. Ayoub:** conceptualization, investigation, funding acquisition, writing – original draft, methodology, validation, visualization, writing – review and editing, software, formal analysis, data curation, resources, project administration. **Hiam Chemaitelly:** data curation, software, writing – review and editing, formal analysis, resources. **Laith J. Abu‐Raddad:** conceptualization, investigation, funding acquisition, writing – original draft, methodology, validation, writing – review and editing, project administration, data curation, supervision, resources.

## Ethics Statement

This study was approved by the Weill Cornell Medicine‐Qatar and Hamad Medical Corporation Institutional Review Boards.

## Consent

The authors have nothing to report.

## Conflicts of Interest

The authors declare no conflicts of interest.

## Supporting information


**Table S1:** Strengthening the Reporting of Observational Studies in Epidemiology (STROBE) checklist for cohort studies.
**Table S2:** Baseline characteristics of the cohort study population.
**Figure S1:** Age‐specific Healthcare Access and Quality (HAQ) Index ratios for each country across the World Health Organization regions. The age‐specific HAQ indices were provided by the Global Burden of Disease study [22].
**Figure S2:** Sensitivity analysis. Comparison between COVID‐19 death estimates from the main analysis and those from the sensitivity analysis, where Qatar's Healthcare Access and Quality (HAQ) Index is used as a reference instead of the highest global HAQ Index.
**Figure S3:** Sensitivity analysis. Comparison of COVID‐19 death estimates from the main analysis with those from the sensitivity analysis, in which the age‐stratified Healthcare Access and Quality (HAQ) Index ratio was either square‐rooted or inflated by adding its natural logarithm, as indicated in the figure legend.

## Data Availability

The dataset of this study is available from the Qatar Ministry of Public Health, but restrictions apply to the availability of these data, which were used under restricted license for the current study, and so are not publicly available. Data are, however, available through a direct application for data access to Her Excellency the Minister of Public Health (https://www.moph.gov.qa/english/OurServices/eservices/Pages/Governmental‐Health‐Communication‐Center.aspx). Aggregate data are included in this published article and the Supporting Information.
