# Supplementary material for: Global Estimates of COVID‐19 Morbidity and Mortality: A Cohort Study and Mathematical Model Analysis
Source: Influenza Other Respir Viruses. 2025 Dec 1;19(12):e70154. doi: 10.1111/irv.70154 (PMC12981520; doi:10.1111/irv.70154)
Supplement: Supplementary file 1 — Table S1: Strengthening the Reporting of Observational Studies in Epidemiology (STROBE) checklist for cohort studies. Table S2: Baseline characteristics of the cohort study population. Figure S1: Age‐specific Healthcare Access and Quality (HAQ) Index ratios for each country across the World Health Organization regions. The age‐specific HAQ indices were provided by the Global Burden of Disease study [22]. Figure S2: Sensitivity analysis. Comparison between COVID‐19 death estimates from the main analysis and those from the sensitivity analysis, where Qatar's Healthcare Access and Quality (HAQ) Index is used as a reference instead of the highest global HAQ Index. Figure S3: Sensitivity analysis. Comparison of COVID‐19 death estimates from the main analysis with those from the sensitivity analysis, in which the age‐stratified Healthcare Access and Quality (HAQ) Index ratio was either square‐rooted or inflated by adding its natural logarithm, as indicated in the figure legend. [file IRV-19-e70154-s001.docx]

**Supplementary Appendix**

**Table of Contents**

[**Section S1. Study population and data sources** 2](#_Toc206525884)

[**Section S2. Laboratory methods and variant ascertainment** 5](#_Toc206525885)

[**Real-time reverse-transcription polymerase chain reaction testing** 5](#_Toc206525886)

[**Rapid antigen testing** 5](#_Toc206525887)

[**Classification of infections by variant type** 6](#_Toc206525888)

[**Section S3. COVID-19 severity, criticality, and fatality classification.** 7](#_Toc206525889)

[**Severe COVID-19** 7](#_Toc206525890)

[**Critical COVID-19** 8](#_Toc206525891)

[**Fatal COVID-19** 8](#_Toc206525892)

[**Table S1.** Strengthening the Reporting of Observational Studies in Epidemiology (STROBE) checklist for cohort studies. 9](#_Toc206525893)

[**Table S2.** Baseline characteristics of the cohort study population. 11](#_Toc206525894)

[**Figure S1.** Age-specific Healthcare Access and Quality (HAQ) Index ratios for each country across the World Health Organization regions. The age-specific HAQ indices were provided by the Global Burden of Disease study [22]. 12](#_Toc206525895)

[**Figure S2.** Sensitivity analysis. Comparison between COVID-19 death estimates from the main analysis and those from the sensitivity analysis, where Qatar's Healthcare Access and Quality (HAQ) Index is used as a reference instead of the highest global HAQ Index. 13](#_Toc206525896)

[**Figure S3.** Sensitivity analysis. Comparison of COVID-19 death estimates from the main analysis with those from the sensitivity analysis, in which the age-stratified Healthcare Access and Quality (HAQ) Index ratio was either square-rooted or inflated by adding its natural logarithm, as indicated in the figure legend. 14](#_Toc206525897)

[**References** 15](#_Toc206525898)

# **Section** **S1.** **Study population and data sources**

Qatar's national and universal public healthcare system uses the Cerner-system advanced digital health platform to track all electronic health record encounters of each individual in the country, including all citizens and residents registered in the national and universal public healthcare system. Registration in the public healthcare system is mandatory for citizens and residents.

The databases analyzed in this study are data-extract downloads from the Cerner-system that have been implemented on a regular weekly schedule since the onset of pandemic by the Business Intelligence Unit at Hamad Medical Corporation (HMC). HMC is the national public healthcare provider in Qatar. At every download all severe acute respiratory syndrome coronavirus 2 (SARS-CoV-2) tests, coronavirus disease 2019 (COVID-19) vaccinations, hospitalizations related to COVID-19, and all death records regardless of cause are provided to the authors through .csv files. These databases have been analyzed throughout the pandemic not only for study-related purposes, but also to provide policymakers with summary data and analytics to inform the national response.

Every health encounter in the Cerner-system is linked to an individual through the HMC Number, which serves as a unique identifier that links all records for this individual at the national level. Databases were merged and analyzed using the HMC Number to link all records pertaining to testing, vaccinations, hospitalizations, and deaths. All deaths in Qatar are recorded by the public healthcare system. All COVID-19-related healthcare was provided exclusively in the public healthcare system. No private entity was permitted to provide COVID-19-related hospitalization. COVID-19 vaccination was also provided only through the public healthcare system. These health records were tracked throughout the COVID-19 pandemic using the Cerner system. This system has been implemented in 2013, before the onset of the pandemic. This pre-established system ensured that we had access to comprehensive health records related to this study for both citizens and residents throughout the entire pandemic, allowing us to follow each person over time.

Demographic details for every HMC Number (individual) such as sex, age, and nationality are collected upon issuing of the universal health card, based on the Qatar Identity Card, which is a mandatory requirement by the Ministry of Interior to every citizen and resident in the country. Data extraction from the Qatar Identity Card to the digital health platform is performed electronically through scanning techniques.

All SARS-CoV-2 testing in any facility in Qatar is tracked nationally in one database, the national testing database. This database covers all testing throughout the country, whether in public or private facilities. Every polymerase chain reaction (PCR) test and a proportion of the facility-based rapid antigen tests conducted in Qatar, regardless of location or setting, are classified on the basis of symptoms and the reason for testing, such as the presence of clinical symptoms, contact tracing, participation in surveys or random testing campaigns, individual requests for testing, routine healthcare testing, pre-travel requirements, at the point of entry into the country, or any other relevant reasons for testing.

Before November 1, 2022, SARS-CoV-2 testing in Qatar was performed extensively with about 5% of the population were tested every week [1]. Based on the distribution of the reason for testing up to November 1, 2022, most of the tests in Qatar were conducted for routine reasons, such as travel-related purposes, and about 75% of infections were diagnosed not because of presence of symptoms [1, 2]. Starting from November 1, 2022, testing for SARS-CoV-2 was substantially reduced, but still close to 1% of the population are being tested every week [2]. This study factored all SARS-CoV-2-related testing included in the national testing database over the duration of follow-up.

Qatar launched its COVID-19 vaccination program in December 2020, employing mRNA vaccines and prioritizing individuals based on coexisting conditions and age criteria [2, 3]. COVID-19 vaccination was provided free of charge, regardless of citizenship or residency status, and was nationally tracked [2, 3].

# **Section S2. Laboratory methods and variant ascertainment**

## **Real-time reverse-transcription polymerase chain reaction testing**

Nasopharyngeal and/or oropharyngeal swabs were collected for PCR testing and placed in Universal Transport Medium (UTM). Aliquots of UTM were: 1) extracted on KingFisher Flex (Thermo Fisher Scientific, USA), MGISP-960 (MGI, China), or ExiPrep 96 Lite (Bioneer, South Korea) followed by testing with real-time reverse-transcription PCR (RT-qPCR) using TaqPath COVID-19 Combo Kits (Thermo Fisher Scientific, USA) on an ABI 7500 FAST (Thermo Fisher Scientific, USA); 2) tested directly on the Cepheid GeneXpert system using the Xpert Xpress SARS-CoV-2 (Cepheid, USA); or 3) loaded directly into a Roche cobas 6800 system and assayed with the cobas SARS-CoV-2 Test (Roche, Switzerland). The first assay targets the viral S, N, and ORF1ab gene regions. The second targets the viral N and E-gene regions, and the third targets the ORF1ab and E-gene regions.

All PCR testing was conducted at the Hamad Medical Corporation Central Laboratory or Sidra Medicine Laboratory, following standardized protocols.

## **Rapid antigen testing**

SARS-CoV-2 antigen tests were performed on nasopharyngeal swabs using one of the following lateral flow antigen tests: Panbio COVID-19 Ag Rapid Test Device (Abbott, USA); SARS-CoV-2 Rapid Antigen Test (Roche, Switzerland); Standard Q COVID-19 Antigen Test (SD Biosensor, Korea); or CareStart COVID-19 Antigen Test (Access Bio, USA). All antigen tests were performed point-of-care according to each manufacturer's instructions at public or private hospitals and clinics throughout Qatar with prior authorization and training by the Ministry of Public Health (MOPH). Antigen test results were electronically reported to the MOPH in real time using the Antigen Test Management System which is integrated with the national Coronavirus Disease 2019 (COVID-19) database.

## **Classification of infections by variant type**

Surveillance for SARS-CoV-2 variants in Qatar is based on viral genome sequencing and multiplex RT-qPCR variant screening [4] of weekly collected random positive clinical samples [2, 5-9], complemented by deep sequencing of wastewater samples [7, 10, 11]. Further details on the viral genome sequencing and multiplex RT-qPCR variant screening throughout the SARS-CoV-2 waves in Qatar can be found in previous publications [1, 2, 5-9, 12-19].

# **Section S3. COVID-19 severity, criticality, and fatality classification.**

Classification of COVID-19 case severity (acute-care hospitalizations) [20], criticality (intensive-care-unit hospitalizations) [20], and fatality [21] followed World Health Organization (WHO) guidelines. Assessments were made by trained medical personnel independent of study investigators and using individual chart reviews, as part of a national protocol applied to every hospitalized COVID-19 patient. Each hospitalized COVID-19 patient underwent an infection severity assessment every three days until discharge or death. We classified individuals who progressed to severe, critical, or fatal COVID-19 between the time of the documented infection and the end of the study based on their worst outcome, starting with death [21], followed by critical disease [20], and then severe disease [20].

## **Severe COVID-19**

Severe COVID-19 disease was defined per WHO classification as a SARS-CoV-2 infected person with "oxygen saturation of <90% on room air, and/or respiratory rate of >30 breaths/minute in adults and children >5 years old (or ≥60 breaths/minute in children <2 months old or ≥50 breaths/minute in children 2-11 months old or ≥40 breaths/minute in children 1–5 years old), and/or signs of severe respiratory distress (accessory muscle use and inability to complete full sentences, and, in children, very severe chest wall indrawing, grunting, central cyanosis, or presence of any other general danger signs)" [20]. Detailed WHO criteria for classifying Severe acute respiratory syndrome coronavirus 2 (SARS-CoV-2) infection severity can be found in the WHO technical report [20].

## **Critical COVID-19**

Critical COVID-19 disease was defined per WHO classification as a SARS-CoV-2 infected person with "acute respiratory distress syndrome, sepsis, septic shock, or other conditions that would normally require the provision of life sustaining therapies such as mechanical ventilation (invasive or non-invasive) or vasopressor therapy" [20]. Detailed WHO criteria for classifying SARS-CoV-2 infection criticality can be found in the WHO technical report [20].

## **Fatal COVID-19**

COVID-19 death was defined per WHO classification as "a death resulting from a clinically compatible illness, in a probable or confirmed COVID-19 case, unless there is a clear alternative cause of death that cannot be related to COVID-19 disease (e.g. trauma). There should be no period of complete recovery from COVID-19 between illness and death. A death due to COVID-19 may not be attributed to another disease (e.g. cancer) and should be counted independently of preexisting conditions that are suspected of triggering a severe course of COVID-19". Detailed WHO criteria for classifying COVID-19 death can be found in the WHO technical report [21].

# **Table S1.** Strengthening the Reporting of Observational Studies in Epidemiology (STROBE) checklist for cohort studies.

|  | Item No | Recommendation | Main Text page |
| --- | --- | --- | --- |
| **Title and abstract** | 1 | (*a*) Indicate the study’s design with a commonly used term in the title or the abstract | Title & Abstract |
|  |  | (*b*) Provide in the abstract an informative and balanced summary of what was done and what was found | Abstract |
| Introduction | | | |
| Background/rationale | 2 | Explain the scientific background and rationale for the investigation being reported | Introduction |
| Objectives | 3 | State specific objectives, including any prespecified hypotheses | Introduction |
| Methods | | | |
| Study design | 4 | Present key elements of study design early in the paper | Methods (‘Cohort study analysis’) |
| Setting | 5 | Describe the setting, locations, and relevant dates, including periods of recruitment, exposure, follow-up, and data collection | Methods (‘Data sources’ in ‘Cohort study analysis’) & Section S1 in Supplementary Appendix |
| Participants | 6 | (*a*) Give the eligibility criteria, and the sources and methods of selection of participants. Describe methods of follow-up | Methods (‘Data sources’, ‘Severe, critical, and fatal COVID-19’, & ‘Cohort follow-up’ in ‘Cohort study analysis’) |
|  |  | (*b*) For matched studies, give matching criteria and number of exposed and unexposed |  |
| Variables | 7 | Clearly define all outcomes, exposures, predictors, potential confounders, and effect modifiers. Give diagnostic criteria, if applicable | Methods ( ‘Cohort follow-up’ & ‘Statistical analysis’ in ‘Cohort study analysis’), & Table S2 & Sections S1-S3 in Supplementary Appendix |
| Data sources/ measurement | 8* | For each variable of interest, give sources of data and details of methods of assessment (measurement). Describe comparability of assessment methods if there is more than one group | Methods (‘Cohort study analysis’), & Table S2 & Sections S1-S3 in Supplementary Appendix |
| Bias | 9 | Describe any efforts to address potential sources of bias | Methods (‘Severe, critical, and fatal COVID-19’ in ‘Cohort study analysis’) & Section S3 in Supplementary Appendix |
| Study size | 10 | Explain how the study size was arrived at | Methods (‘Data sources’ & ‘Cohort follow-up’ in ‘Cohort study analysis’) |
| Quantitative variables | 11 | Explain how quantitative variables were handled in the analyses. If applicable, describe which groupings were chosen and why | Methods (‘Statistical analysis’) & Table S2 in Supplementary Appendix |
| Statistical methods | 12 | (*a*) Describe all statistical methods, including those used to control for confounding | Methods (‘Statistical analysis’) |
|  |  | (*b*) Describe any methods used to examine subgroups and interactions | Methods (‘Statistical analysis’) |
|  |  | (*c*) Explain how missing data were addressed | Not applicable, see Methods (‘Data sources’ in ‘Cohort study analysis’) & Section S1 in Supplementary Appendix |
|  |  | (*d*) If applicable, explain how loss to follow-up was addressed | Not applicable, see Methods (‘Data sources’ in ‘Cohort study analysis’) & Section S1 in Supplementary Appendix |
|  |  | (*e*) Describe any sensitivity analyses | Methods (‘Sensitivity analyses’ in ‘Mathematical modelling analysis’) |
| Results | | |  |
| Participants | 13* | (a) Report numbers of individuals at each stage of study—eg numbers potentially eligible, examined for eligibility, confirmed eligible, included in the study, completing follow-up, and analysed | Methods (‘Cohort follow-up’ in ‘Cohort study analysis’) & Table S2 in Supplementary Appendix |
|  |  | (b) Give reasons for non-participation at each stage |  |
|  |  | (c) Consider use of a flow diagram |  |
| Descriptive data | 14 | (a) Give characteristics of study participants (eg demographic, clinical, social) and information on exposures and potential confounders | Table S2 in Supplementary Appendix |
|  |  | (b) Indicate number of participants with missing data for each variable of interest | Not applicable, see Methods (‘Data sources’ in ‘Cohort study analysis’) & Section S1 in Supplementary Appendix |
|  |  | (c) Summarise follow-up time (eg, average and total amount) | Methods (‘Cohort follow-up’ in ‘Cohort study analysis’) |
| Outcome data | 15 | Report numbers of outcome events or summary measures over time | Results (‘Cohort analysis’, ‘Estimates for severe, critical, or fatal COVID-19 cases and COVID-19 deaths’, & ‘Number and proportion of cases by pandemic phase’), & Figures 1-4 |
| Main results | 16 | (a) Give unadjusted estimates and, if applicable, confounder-adjusted estimates and their precision (eg, 95% confidence interval). Make clear which confounders were adjusted for and why they were included | Results (‘Cohort analysis’, ‘Estimates for severe, critical, or fatal COVID-19 cases and COVID-19 deaths’, & ‘Number and proportion of cases by pandemic phase’), & Figures 1-4 |
|  |  | (b) Report category boundaries when continuous variables were categorized | Figures 1-4, & Figure S1 & Table S2 in Supplementary Appendix |
|  |  | (c) If relevant, consider translating estimates of relative risk into absolute risk for a meaningful time period | Not applicable |
| Other analyses | 17 | Report other analyses done—eg analyses of subgroups and interactions, and sensitivity analyses | Results (‘Comparison between model-estimated and reported COVID-19 deaths’ & ‘Sensitivity analyses for COVID-19 deaths’), Figure 5 & Figures S2 and S3 in Supplementary Appendix |
| Discussion | | | |
| Key results | 18 | Summarise key results with reference to study objectives | Discussion, paragraphs 1-9 |
| Limitations | 19 | Discuss limitations of the study, taking into account sources of potential bias or imprecision. Discuss both direction and magnitude of any potential bias | Discussion, paragraphs 10-17 |
| Interpretation | 20 | Give a cautious overall interpretation of results considering objectives, limitations, multiplicity of analyses, results from similar studies, and other relevant evidence | Discussion, paragraph 18 |
| Generalisability | 21 | Discuss the generalisability (external validity) of the study results | Discussion, paragraphs 15-17 |
| Other information | | | |
| Funding | 22 | Give the source of funding and the role of the funders for the present study and, if applicable, for the original study on which the present article is based | Funding |

# **Table S2.** Baseline characteristics of the cohort study population.

| **Characteristics** | **National Qatari cohort** |
| --- | --- |
|  | **N=312,876** |
| Median age (interquartile range)—years | 23 (11-39) |
| Age group |  |
| 0-9 years | 74,298 (23.8) |
| 10-19 years | 67,823 (21.7) |
| 20-29 years | 53,295 (17.0) |
| 30-39 years | 42,989 (13.7) |
| 40-49 years | 30,215 (9.7) |
| 50-59 years | 22,374 (7.2) |
| 60-69 years | 13,194 (4.2) |
| 70+ years | 8,688 (2.8) |
| Sex |  |
| Male | 154,007 (49.2) |
| Female | 158,869 (50.8) |
| Number of coexisting medical conditions |  |
| None | 179,471 (57.4) |
| 1 | 71,276 (22.8) |
| 2 | 28,165 (9.0) |
| 3 | 12,574 (4.0) |
| 4 | 8,453 (2.7) |
| 5 | 5,673 (1.8) |
| 6+ | 7,264 (2.3) |

# **Figure S1.** Age-specific Healthcare Access and Quality (HAQ) Index ratios for each country across the World Health Organization regions. The age-specific HAQ indices were provided by the Global Burden of Disease study [22].


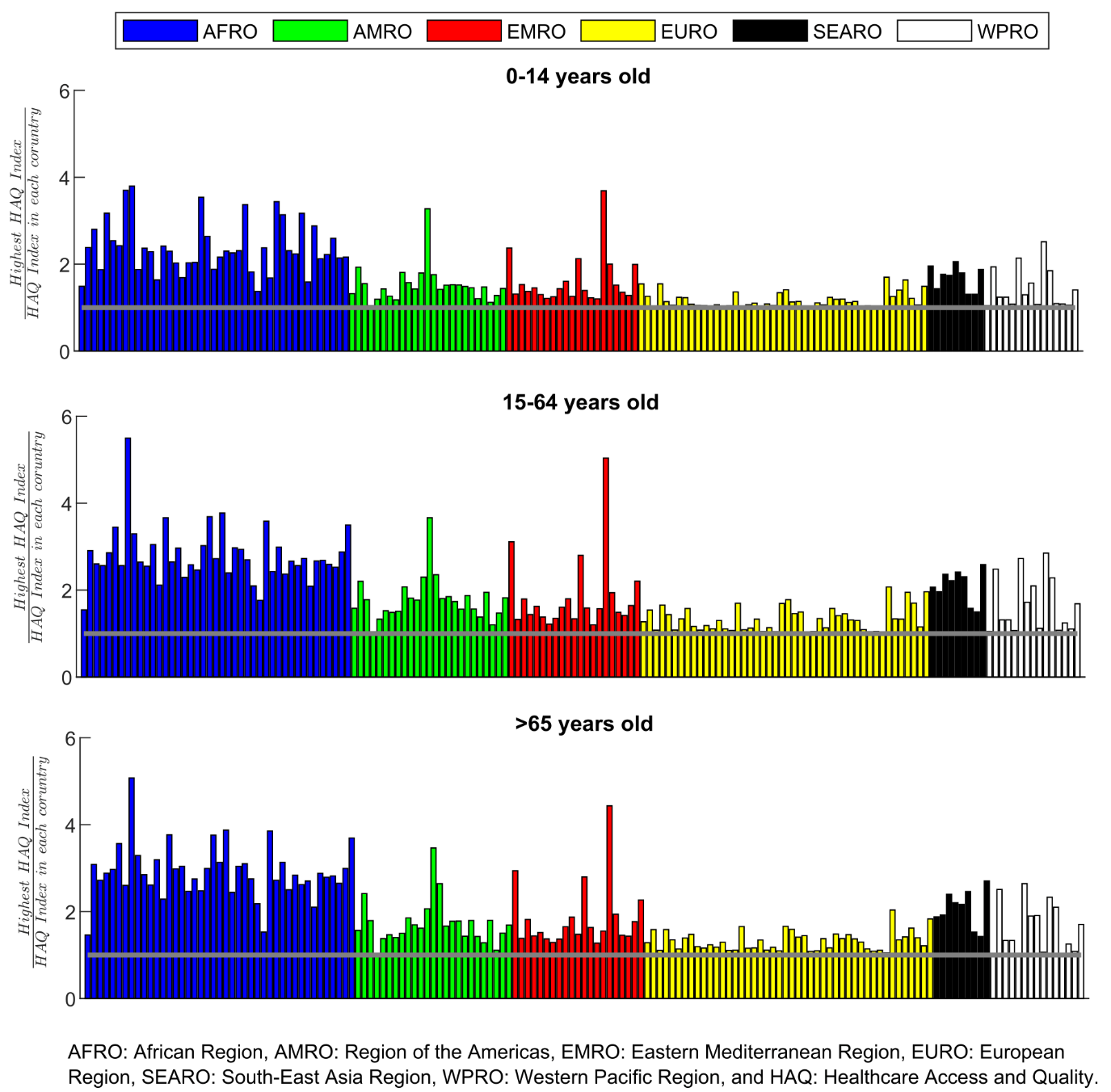


# **Figure S2.** Sensitivity analysis. Comparison between COVID-19 death estimates from the main analysis and those from the sensitivity analysis, where Qatar's Healthcare Access and Quality (HAQ) Index is used as a reference instead of the highest global HAQ Index.


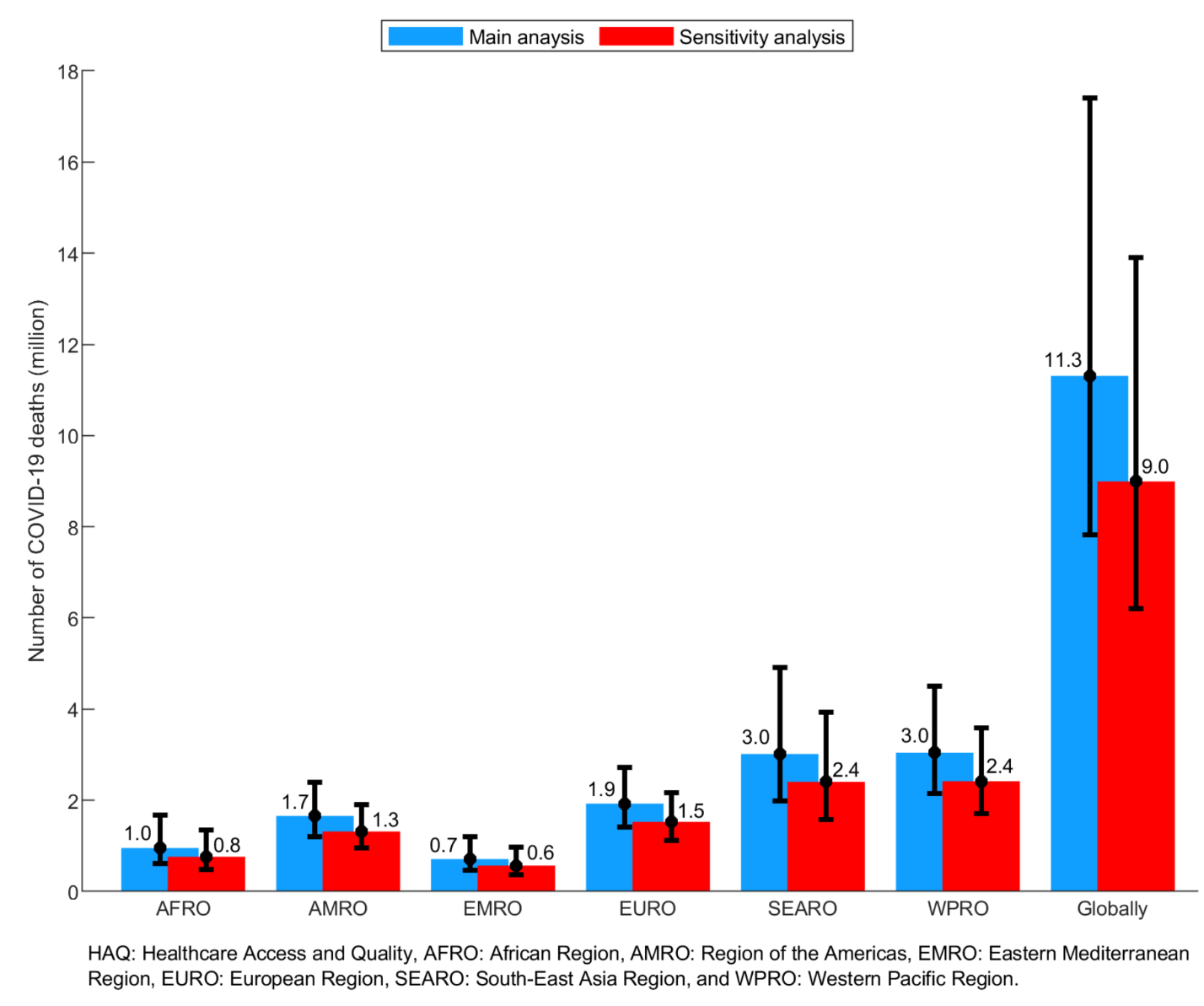


# **Figure S3.** Sensitivity analysis. Comparison of COVID-19 death estimates from the main analysis with those from the sensitivity analysis, in which the age-stratified Healthcare Access and Quality (HAQ) Index ratio was either square-rooted or inflated by adding its natural logarithm, as indicated in the figure legend.


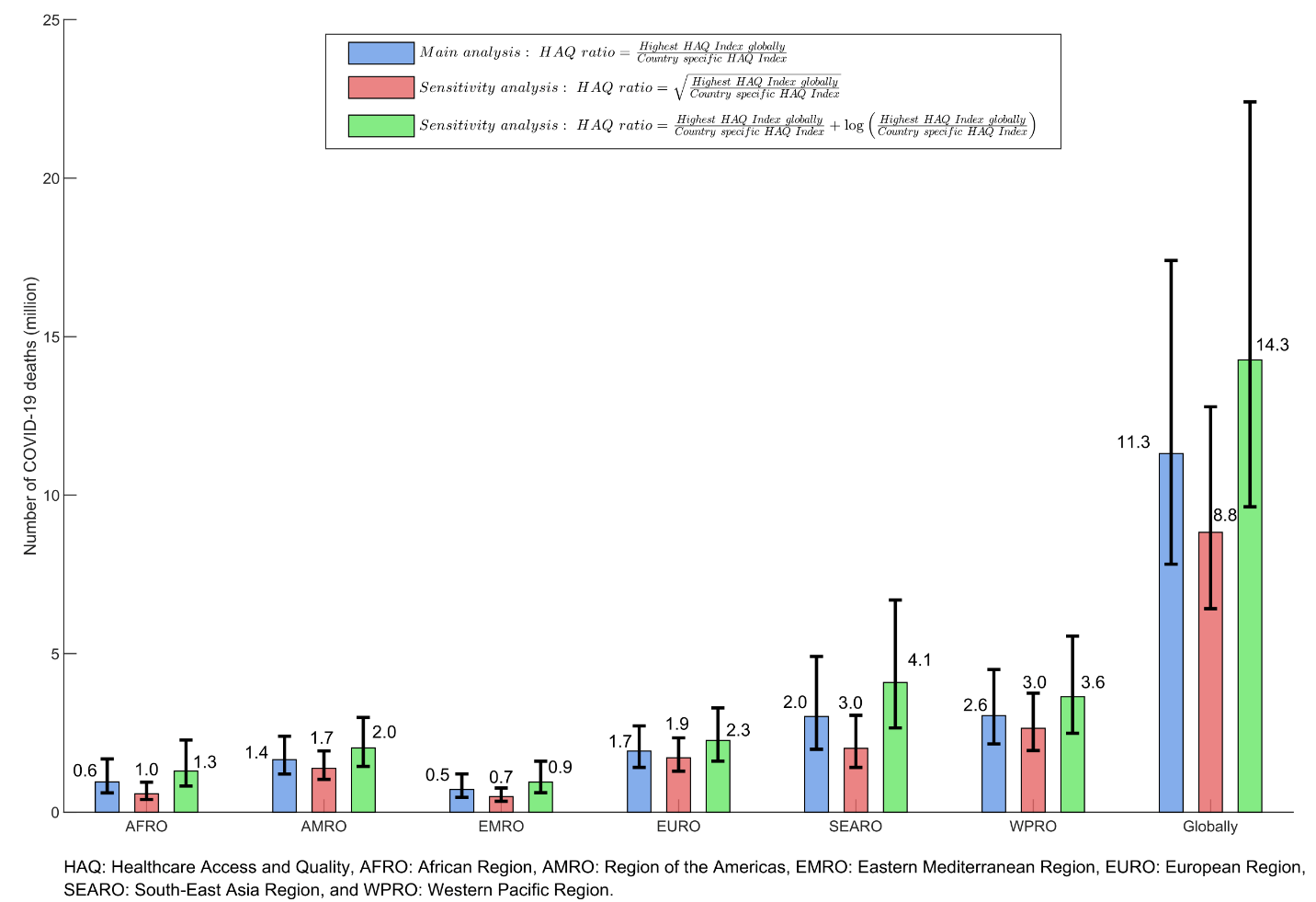


# **References**

1. Altarawneh, H.N., et al., *Effects of Previous Infection and Vaccination on Symptomatic Omicron Infections.* N Engl J Med, 2022. **387**(1): p. 21-34.

2. Chemaitelly, H., et al., *Waning of BNT162b2 Vaccine Protection against SARS-CoV-2 Infection in Qatar.* N Engl J Med, 2021. **385**(24): p. e83.

3. Abu-Raddad, L.J., et al., *Effectiveness of mRNA-1273 and BNT162b2 Vaccines in Qatar.* N Engl J Med, 2022. **386**(8): p. 799-800.

4. Vogels, C., J. Fauver, and N. Grubaugh, *Multiplexed RT-qPCR to screen for SARS-COV-2 B.1.1.7, B.1.351, and P.1 variants of concern V.3. dx.doi.org/10.17504/protocols.io.br9vm966.* 2021(June 6, 2021).

5. Abu-Raddad, L.J., et al., *Effectiveness of the BNT162b2 Covid-19 Vaccine against the B.1.1.7 and B.1.351 Variants.* N Engl J Med, 2021. **385**(2): p. 187-189.

6. Chemaitelly, H., et al., *mRNA-1273 COVID-19 vaccine effectiveness against the B.1.1.7 and B.1.351 variants and severe COVID-19 disease in Qatar.* Nat Med, 2021. **27**(9): p. 1614-1621.

7. National Project of Surveillance for Variants of Concern and Viral Genome Sequencing. *Qatar viral genome sequencing data. Data on randomly collected samples.* [*https://www.gisaid.org/phylodynamics/global/nextstrain/*](https://www.gisaid.org/phylodynamics/global/nextstrain/). 2021; Available from: <https://www.gisaid.org/phylodynamics/global/nextstrain/>.

8. Benslimane, F.M., et al., *One Year of SARS-CoV-2: Genomic Characterization of COVID-19 Outbreak in Qatar.* Front Cell Infect Microbiol, 2021. **11**: p. 768883.

9. Hasan, M.R., et al., *Real-Time SARS-CoV-2 Genotyping by High-Throughput Multiplex PCR Reveals the Epidemiology of the Variants of Concern in Qatar.* Int J Infect Dis, 2021. **112**: p. 52-54.

10. Saththasivam, J., et al., *COVID-19 (SARS-CoV-2) outbreak monitoring using wastewater-based epidemiology in Qatar.* Sci Total Environ, 2021. **774**: p. 145608.

11. El-Malah, S.S., et al., *Application of human RNase P normalization for the realistic estimation of SARS-CoV-2 viral load in wastewater: A perspective from Qatar wastewater surveillance.* Environ Technol Innov, 2022. **27**: p. 102775.

12. Abu-Raddad, L.J., et al., *Effect of mRNA Vaccine Boosters against SARS-CoV-2 Omicron Infection in Qatar.* N Engl J Med, 2022. **386**(19): p. 1804-1816.

13. Tang, P., et al., *BNT162b2 and mRNA-1273 COVID-19 vaccine effectiveness against the SARS-CoV-2 Delta variant in Qatar.* Nat Med, 2021. **27**(12): p. 2136-2143.

14. Altarawneh, H.N., et al., *Protection against the Omicron Variant from Previous SARS-CoV-2 Infection.* N Engl J Med, 2022. **386**(13): p. 1288-1290.

15. Chemaitelly, H., et al., *Duration of mRNA vaccine protection against SARS-CoV-2 Omicron BA.1 and BA.2 subvariants in Qatar.* Nat Commun, 2022. **13**(1): p. 3082.

16. Qassim, S.H., et al., *Effects of BA.1/BA.2 subvariant, vaccination and prior infection on infectiousness of SARS-CoV-2 omicron infections.* J Travel Med, 2022. **29**(6).

17. Altarawneh, H.N., et al., *Protective Effect of Previous SARS-CoV-2 Infection against Omicron BA.4 and BA.5 Subvariants.* N Engl J Med, 2022. **387**(17): p. 1620-1622.

18. Chemaitelly, H., et al., *Protection against Reinfection with the Omicron BA.2.75 Subvariant.* N Engl J Med, 2023. **388**(7): p. 665-667.

19. Chemaitelly, H., et al., *Protection of natural infection against reinfection with SARS-CoV-2 JN.1 variant.* medRxiv, 2024: p. 2024.02.22.24303193.

20. World Health Organization (WHO), *Living guidance for clinical management of COVID-19. Aavailable from:* [*https://www.who.int/publications/i/item/WHO-2019-nCoV-clinical-2021-2*](https://www.who.int/publications/i/item/WHO-2019-nCoV-clinical-2021-2)*. Accessed on: February 27, 2023.* 2021.

21. World Health Organization (WHO), *International Guidelines for Certification and Classification (Coding) of COVID-19 as Cause of Death. Available from:* [*https://www.who.int/publications/m/item/international-guidelines-for-certification-and-classification-(coding)-of-covid-19-as-cause-of-death*](https://www.who.int/publications/m/item/international-guidelines-for-certification-and-classification-(coding)-of-covid-19-as-cause-of-death)*. Accessed on: February 27, 2023.* 2020.

22. Haakenstad, A., et al., *Assessing performance of the Healthcare Access and Quality Index, overall and by select age groups, for 204 countries and territories, 1990–2019: a systematic analysis from the Global Burden of Disease Study 2019.* The Lancet global health, 2022. **10**(12): p. e1715-e1743.
